# Membrane Partitioning of Anionic, Ligand-Coated Nanoparticles Is Accompanied by Ligand Snorkeling, Local Disordering, and Cholesterol Depletion

**DOI:** 10.1371/journal.pcbi.1003917

**Published:** 2014-12-04

**Authors:** Paraskevi Gkeka, Panagiotis Angelikopoulos, Lev Sarkisov, Zoe Cournia

**Affiliations:** 1 Biomedical Research Foundation, Academy of Athens, Athens, Greece; 2 Computational Science and Engineering Laboratory, Institute of Computational Science, D-MAVT, ETH Zurich, Switzerland; 3 Institute for Materials and Processes, School of Engineering, The University of Edinburgh, Edinburgh, United Kingdom; Max Planck Institute for Biophysical Chemistry, Germany

## Abstract

Intracellular uptake of nanoparticles (NPs) may induce phase transitions, restructuring, stretching, or even complete disruption of the cell membrane. Therefore, NP cytotoxicity assessment requires a thorough understanding of the mechanisms by which these engineered nanostructures interact with the cell membrane. In this study, extensive Coarse-Grained Molecular Dynamics (MD) simulations are performed to investigate the partitioning of an anionic, ligand-decorated NP in model membranes containing dipalmitoylphosphatidylcholine (DPPC) phospholipids and different concentrations of cholesterol. Spontaneous fusion and translocation of the anionic NP is not observed in any of the 10-µs unbiased MD simulations, indicating that longer timescales may be required for such phenomena to occur. This picture is supported by the free energy analysis, revealing a considerable free energy barrier for NP translocation across the lipid bilayer. 5-µs unbiased MD simulations with the NP inserted in the bilayer core reveal that the hydrophobic and hydrophilic ligands of the NP surface rearrange to form optimal contacts with the lipid bilayer, leading to the so-called snorkeling effect. Inside cholesterol-containing bilayers, the NP induces rearrangement of the structure of the lipid bilayer in its vicinity from the liquid-ordered to the liquid phase spanning a distance almost twice its core radius (8–10 nm). Based on the physical insights obtained in this study, we propose a mechanism of cellular anionic NPpartitioning, which requires structural rearrangements of both the NP and the bilayer, and conclude that the translocation of anionic NPs through cholesterol-rich membranes must be accompanied by formation of cholesterol-lean regions in the proximity of NPs.

## Author Summary

The increasing applications of nanotechnology in medicine rely on the fact that engineered nanomaterials, such as diagnostic and therapeutic nanoparticles, are able to translocate across the cellular membrane and reach their site of action without toxic effects. One of the first steps into assessing the NP cytotoxicity requires a thorough understanding of the nanoparticle-membrane interaction mechanism. We have computationally investigated, using unprecedented spatiotemporal effort, the structure and dynamics of anionic NP partitioning in explicit cholesterol-containing membranes. Our results show that NP partitioning in the membrane is accompanied by the rearrangement of the NP surface ligands and causes the re-organization of the lipids and cholesterol in its vicinity. In this context, our study is an early step towards novel strategies for tailored decoration of NPs aiming to selectively target specific cells based on their cholesterol content.

## Introduction

Understanding the interaction mechanisms between nanoparticles (NPs) and cell membranes is of critical importance for their use in medical applications [1]–[5]. In these applications, engineered nanostructures are required to contact target cells without damaging essential tissues. The ability of NPs to reach intracellular compartments depends on their morphology and surface chemistry as well as on environmental factors such as cell type, pH, etc. A number of experimental (for a review see [6]) and simulation [7]–[15] studies focused on the effect of NP physico-chemical properties on their interaction with membranes and other liquid/liquid interfaces. In a striking example, Stellacci and co-workers [16] prepared gold NPs (AuNPs) coated with hydrophobic and hydrophilic ligands, which assemble into well-defined striped domains depending on ligand composition. Subsequent in vivo studies on cells suggested both endocytotic and direct translocation mechanisms for striped NPs, whereas NPs with random hydrophilic surfaces could translocate only via endocytosis [17]. Recently, the existence of the striped domains on the surface of these NPs has been challenged by an alternative interpretation of the experimental results, prompting new studies to understand the mechanisms of interactions between NPs and biological membranes [18].

Why do certain NPs easily translocate through biological membranes and others do not? Answering this question would enable us to assess cytotoxicity of various nanostructures and harvest their properties for tailored applications. Unfortunately, current toxicological knowledge about NPs is limited and does not allow for a complete understanding of the effect of nanostructure morphology, composition, and aggregation-dependent interactions with biological systems. Molecular simulations can help rationalize experimental findings by providing a microscopic-level description of the NP-membrane interactions. Such a microscopic-level picture could lead to the creation of predictive models for estimating NP cytotoxicity. This, however, is an immensely challenging task.

Firstly, in vivo processes are too complex to be directly considered in molecular simulations. A cell membrane is a multilayer entity featuring a complex composition of lipids, proteins, and other components, while its environment is a complex solution containing ions, proteins and other species. Therefore, one must resort to simplified models to assess and decouple the influence of various factors on NP cytotoxicity. Secondly, the processes of interest may take place on spatiotemporal scales, which are difficult to access with atomistic simulations. For this reason, simulation studies of NP-membrane interactions are usually based on Coarse-Grained (CG) models, where a group of several atoms is represented by one effective interaction bead.

Not surprisingly, one of these CG models, MARTINI [19], has been extensively applied to the study of NP-membrane interactions [9]–[11], [15], [20]–[22]. Previously, we aimed to understand the role of hydrophobic and hydrophilic domains in the NP translocation process, assuming that these domains do form and remain intact regardless of the NP environment [15]. Therefore, ligand chains on the NP surface were not modeled explicitly, but were represented with CG beads of hydrophilic and hydrophobic types according to the MARTINI model, arranged on the NP surface forming domains of a particular geometry. It was shown that the free energy barrier for NP translocation across the bilayer could be manipulated by controlling the size of hydrophobic domains, onto which lipid molecules tend to self-assemble. In the same study we considered an NP with ligand chains represented explicitly using the same CG model and although the domains on the NP surface were initially designed to follow a particular pattern (i.e. stripes) NP surface ligand flexibility allowed the ligand chains to re-arrange, resulting in a surface chemistry and geometry significantly different from the designed underlying surface pattern as a response to the environment.

In a different study, Lin et al. modeled the interaction of AuNPs coated with flexible ligands with two different types of lipid bilayers [7]. The systems consisted of ∼1,000 lipids and were simulated for a few nanoseconds in unbiased and biased MD. Based on their biased simulations, the authors found that electrostatic interactions between the charged AuNP ligands and the lipid head groups govern the binding of AuNPs to bilayers and that, upon penetration, defective areas and a water pore are induced in the bilayer, while the lipids close to the NP are considerably disordered.

The importance of surface ligand flexibility has also been studied in two articles by Van Lehn and co-workers [23], [24]. The authors also observed that the initial pattern of ligands on the surface of a AuNP is likely not important in the consequent behavior of the NP as the ligands tend to re-arrange within the lipid bilayer so that their polar/charged heads “snorkel” to the surface. In their work, however, the bilayer was modeled implicitly and therefore, the model could not capture the process of lipid reorganization around the NP. Furthermore, only the free energy difference between the NP in the water phase and in the bilayer core was reported, leaving the intermediate states of the system and dynamics of the process outside of the scope of the study.

The above-mentioned studies highlight the importance of a sufficient level of realism of the model to capture the phenomena of interest. An integral aspect of the membrane complexity that has been so far neglected is the presence of other components in the membrane. In particular, cholesterol can substantially influence membrane physical properties, such as fluidity, and induces the formation of lipid rafts, which play an important role in signal transduction and thus in several diseases [25]. Recently, it was shown that cells from certain cancer tissues contain higher concentrations of cholesterol compared to healthy cells [26]. This variation of the cholesterol concentration can be exploited in accurate targeting of cancer cells in advanced drug delivery strategies.

Herein, we investigate the partitioning of anionic NPs into explicit cholesterol-containing dipalmitoylphosphatidylcholine (DPPC) bilayers using biased and unbiased MD simulations. Our results demonstrate that the timescale of spontaneous NP fusion and translocation exceeds the unbiased MD simulated timescale (10 µs). We show that NP partitioning in the bilayer causes rearrangement of the NP surface ligands to facilitate the “snorkeling” of the charged groups towards the lipid head-groups, while its hydrophobic chains remain buried in the bilayer core. Embedding of an NP into the bilayer also forces lipids and cholesterol to re-organize. Specifically, we observe that cholesterol concentration is lower in the vicinity of the NP, while the bilayer structure is more disordered and corresponds to the liquid phase. We conclude with a discussion of the NP decoration as a tuning parameter controlling NP translocation mechanism through the formation of cholesterol-lean patches.

## Results

### Partitioning mechanism of a striped anionic NP in lipid bilayers

**Figure 1:**
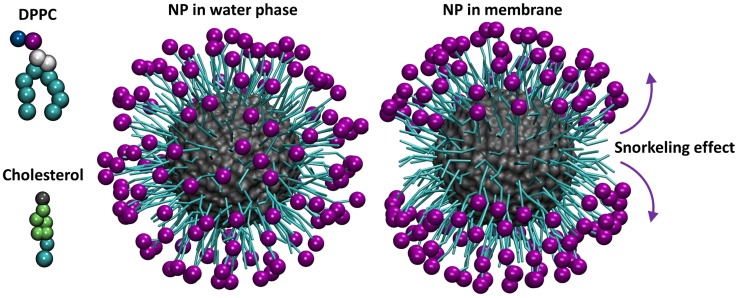
Coarse-grained models of the system components. DPPC molecule (top, left), cholesterol molecule (bottom, left), nanoparticle simulated in the water phase (center), and nanoparticle simulated in a membrane containing 30% cholesterol (right) (final snapshots). The molecules or CG beads are not shown to scale. Colors: Negative beads bearing -1e charge = purple; hydrophobic beads = cyan; positive beads = blue; cholesterol hydroxyl bead = gray; glycerol backbone beads = white; cholesterol sterol body beads = lime. The core of the NP is shown in gray and surface representation, whereas the hydrophobic parts of NP surface ligands are shown in a bead-spring representation. Arrows indicate the tendency of the surface charged ligands to associate with the DPPC head groups and their hydrophobic parts to interact with the hydrophobic core, inducing a snorkeling effect.

We consider an anionic NP with a core diameter of 4.3 nm coated with regular, striped patterns of hydrophobic (octanethiol, OT) and hydrophilic, negatively charged (11-mercapto-1-undecanesulphonate, MUS) domains in a 2∶1 MUS∶OT ratio, inspired by recent studies [17], [24], [27]. In our model, the bilayer, water phase, and NP are modeled with a CG representation using the MARTINI force field (Figure 1) [19]. The NP is coated with surface ligands that are represented explicitly as flexible chains (Figure S1 in Text S1, Supporting Information (SI)). The modeled NP is negatively charged with a charge density of 1.19 e/nm^2^ and total charge of −134. Charged ligands are often used as capping agents on NPs to keep them separated via electrostatic repulsion and prevent their aggregation, which has been linked to cytotoxic effects [28].

In general, the burial of a charge in a low dielectric medium such as the lipid bilayer is associated with a substantial energy penalty. For example, atomistic simulations show that in this process the ion remains solvated and its translocation requires formation of water defects, which is accompanied by barriers of 91.7 kJ/mol for Na+ and 98.8 kJ/mol for Cl− [29]. Given the free energy barrier required for a single ion to translocate across a lipid bilayer, we were intrigued by the recent study of van Lehn et al. [24], who predicted that AuNPs decorated with anionic and hydrophobic ligands should prefer, for the majority of systems they explored, to be located inside the lipid membrane compared to the water phase. Depending on the size of the particle, the free energy change between the water phase and the bilayer core varied between slightly positive values (corresponding to the core of the bilayer not being the favorable location for the NP) to as low as −715 kJ/mol for 3.5 nm 2∶1 MUS∶OT NPs and −1,205 kJ/mol for 4.5 nm 1∶1 MUS∶OT NPs [23]. The magnitude of these free energy minima implies that these specific NPs should be trapped in the core of the bilayer indefinitely, although particles of other sizes studied by the authors and featuring not as deep free energy minima in the bilayer core may actually be able to translocate into the cell interior. The authors argued, that this behavior, being similar to one of a purely hydrophobic NP, is due to the ability of the flexible ligands to rearrange, thus increasing the contact area between the hydrophobic residues and bilayer core.

Therefore, we set out to investigate in more detail the origin of this behavior for the anionic NP translocation by exploring the mechanism of association of the NP with the lipid bilayer. To establish a relationship between the NP structure and its interaction mechanism with membranes of different composition, six different systems were considered: a cholesterol-free bilayer and bilayers containing 10%, 20%, 30%, 40%, and 50% mol. cholesterol, each with approximately 8,000–10,000 lipids in total except for the 50% mol. bilayer, which consisted of 14,000 lipids (the exact system sizes are shown in Table S1 in Text S1). The total simulation time was 10 µs for all systems except the system with 50% mol. cholesterol, where the simulation was performed for 8.5 µs. For the modeling of the systems we employed the MARTINI force field (the details of the simulations are provided in the methodology and Text S1) [19]. The parameters of the force field, including the mapping of the atoms to coarse-grained particles, are calibrated to reproduce the free energy differences for partitioning between polar and apolar phases for several reference species. We note, however, that in general the CG MARTINI force field does not reproduce the water defect formation upon translocation of charged groups across lipid bilayers. For example, unlike in atomistic simulations of charged residues, in CG MARTINI representation these residues tend to lose their hydration shell at 0.7 nm away from the bilayer center, which is mostly due to the first hydration shell being included in the coarse-grained representation of charged MARTINI particles [19]. We estimated that the free energy barrier for the translocation of a sodium ion across a DPPC lipid bilayer is 60 kJ/mol (Figure S2 in Text S1, also see Text S1 for details of the calculation). Although this value is lower than the results from atomistic simulations, it is consistent with other CG simulation studies that report energy barriers of 69.0 kJ/mol and 69.2 kJ/mol for Na^+^ and Cl^−^ translocation, respectively [29]. The barrier for a sodium ion to translocate through a cholesterol-containing bilayer (50% mol.) is even higher (80 kJ/mol, Figure S2 in Text S1); this increase in the free energy barrier is expected as the addition of cholesterol to a fluid phase bilayer decreases the permeability of water and ions [30], [31]. One might conjecture this increase in the energy barrier across cholesterol-containing membranes also for NPs decorated with charged ligands, and go even further to speculate that the details of ligand rearrangement on the surface of NP upon its insertion in the membrane must depend on the composition of the membrane.

Initially, we performed unbiased simulations placing the NP in the water phase 4 nm away from the bilayer surface. Within the first 50 ns of each simulation, the NP partitions at the surface of the bilayer for all different bilayer systems. Figures S3 and S4 in Text S1 show the final simulation snapshots, where the NP adopts a position close to the bilayer-water interface. Within the simulation time we observe no evidence of possible lipid or ligand rearrangement that would propose NP fusion with the bilayer. The number density maps (see Text S1 for the definition) in Figure S4 in Text S1 show no reorganization of the negatively charged end-terminal groups of the NP ligands over the last 500 ns of the simulation.

**Figure 2:**
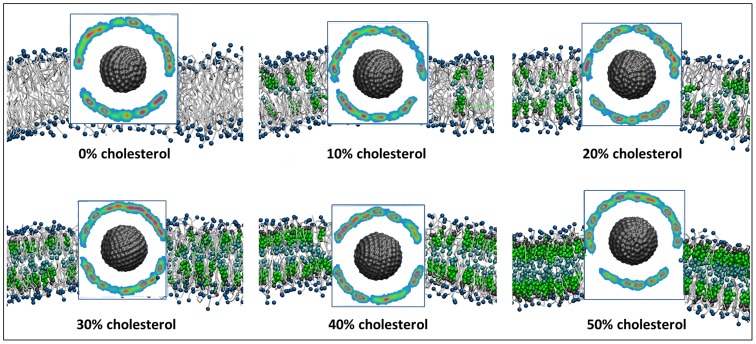
Number density maps of the negatively charged end-terminal groups of the NP ligands. The calculation was performed over the last 500 ns of the simulation for the different systems. The snapshots correspond to the final frame of the simulation and are depicted to indicate the relative position of the NP with respect to the lipid bilayer at the end of the simulation. White corresponds to zero density of the negatively-charged moieties, indicating the snorkeling effect, where charged-end terminal groups orient themselves towards the lipid head groups and outside of the bilayer core. A typical RGB color scale is used to show increasing occupancy. Only the NP core is shown for clarity. The coloring is the same as in Figure 1.

Therefore, to investigate the structure of the NP-bilayer system upon NP partitioning, we performed a series of unbiased 5 µs CG-MD simulations with the NP placed inside the hydrophobic core of preassembled and equilibrated bilayers. After equilibration, in all considered systems, the NP positions itself either in the core of the bilayer or close to the bilayer-water interface (Figure S5 in Text S1). We observe that the hydrophobic ligands on the NP surface ligands rearrange in order to associate with the bilayer interior, maximizing hydrophobic and minimizing polar contacts. At the same time, the negatively charged MUS termini form salt bridges with the positively charged choline group of the DPPC molecules inducing the so-called “snorkeling effect” (Figure S5 in Text S1). This effect was also observed in the study of Ref. [24]. In Figure 2, the “snorkeling” effect is presented using the number density maps of the negatively charged end-terminal groups of the NP ligands over the last 500 ns of the simulation for each system.

Our observations from both simulation setups (NP initially in water and in the bilayer center) bring to light the microscopic features of the NP-membrane systems described above and most importantly the effect of ligand flexibility in bilayer-NP interactions. It should be noted that the explicit representation of flexible ligands is an important attribute of the system and cannot be neglected as in previous studies, where the NPs were designed as smooth objects with surface patterns [11], [15]. Visual inspection of the MD trajectories indicates that the “hairy” nature of the NP hinders lipid aggregation around it, as the disordered environment of the flexible chains prevents direct access of the hydrophobic lipid tails to the NP surface contrary to what was observed in the case of the smooth NPs [15]. Additionally, the NP anionic surface charges associate with the choline groups of the DPPC bilayer. These features render the “hairy” NP less prone to be incorporated within the bilayer compared to the smooth NPs. Indeed, spontaneous insertion of the specific NP has not been observed in any of our simulations, indicating that longer timescales are required for this process.

### Free energy calculations

**Figure 3:**
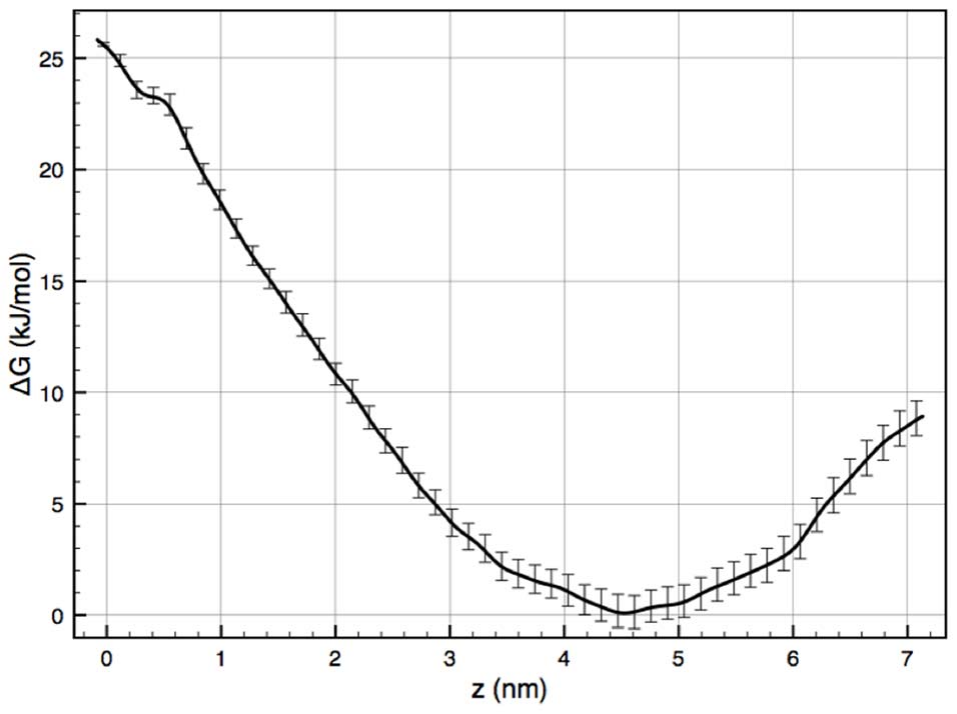
PMF for NP partitioning in a cholesterol-free DPPC lipid bilayer. The error bars represent standard deviations from two independent sets of Umbrella sampling calculations using the bootstrapping technique. Detailed analysis of the PMF convergence is provided in Text S1.

Equilibrium MD simulations did not show spontaneous penetration of the NP into the bilayer. Intuitively, this result should have been expected as the translocation of a highly charged NP from water into the bilayer must entail a substantial energy penalty to bury the exposed anionic heads of the ligands into the hydrophobic medium of the bilayer. To assess the free energy barriers associated with this process and elucidate the underlying molecular mechanisms of interaction, we performed Potential of Mean Force (PMF) calculations. Given the intrinsic complexity of PMF calculations of large and slowly evolving systems, an extensive investigation on the sampling time that is necessary for the convergence of the PMF has been performed and is presented in the SI (Figures S6 and S7 in Text S1). It is interesting to note that the free energy for the insertion of the NP from the water to the core of the bilayer fluctuates only between 27 kJ/mol and 29 kJ/mol between sampling times of 50 up to 400 ns for the cholesterol-free membrane (Figure S7 in Text S1). For the 50% mol. cholesterol system, the fluctuation of the barrier is also small, between 49 kJ/mol and 56 kJ/mol between sampling times of 50 up to 300 ns, however its increasing trend does not allow us to conclude on the convergence of the calculations. The PMF profiles with respect to the distance from the bilayer midline are shown in Figure 3 for the cholesterol-free system and Figure S8 in Text S1 for the 50% mol. cholesterol system.

For the cholesterol-free membrane, an energy minimum can be observed at a distance of ≈4.5 nm from the center of the bilayer. At about 4 nm distance from the bilayer center, the polar ligands on the NP surface interact with the positively charged choline groups of the lipids. The NP has to overcome a barrier of 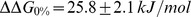, in order to translocate from the bilayer-water interface (minimum) inside the bilayer, indicating a non-spontaneous process on the simulated timescale (Figure 3). In Figure S8 in Text S1, we present the PMF profile calculated from 300 ns per window for the 50% mol. cholesterol membrane. Based on our convergence analysis discussed above, this result should be considered with caution. However, qualitative information provided by comparing the two free energy profiles, i.e. 0% and 50% mol. cholesterol, is consistent with what one would expect: the higher the cholesterol concentration, the more difficult the NP translocation across a membrane.

Although inspired by the same experimental system, the employed models and methodologies are very different in our study and in the article by van Lehn and co-workers, since we use an explicit bilayer, use the MARTINI force field instead of a bespoke force field and for the calculation of the free energy difference we perform Umbrella sampling calculations while van Lehn et al use an approach based on the free energy decomposition [24]. Nevertheless, here we attempt to establish an overarching link to their observations. Some of their systems exhibited zero free energy difference between water phase and the bilayer core for the NP location (and even slightly positive values, depending on the NP size), while the majority of their systems exhibited a substantial free energy minimum in the bilayer core. Our studies reveal positive free energy barrier to NP translocation across the bilayer with the minimum lying at the NP-bilayer interface. The difference in van Lehn's and our results indicate that the free energy penalty is a strong function of whether a charged group is buried inside the bilayer core. The “snorkeling” mechanism allows flexible ligands to avoid this penalty by relocating their charged termini to the surface of the bilayer. The efficiency of this “snorkeling” mechanism should, however, depend on the length of ligands (or, alternatively, on the size of the NP core), their flexibility, and packing. In our model, a fraction of the charged groups remain entangled in the bilayer core, unable to find a pathway to the surface of the bilayer (Figure S5 in Text S1). This entrapment leads to an energy penalty and positive free energy barrier. In some of the systems studied by van Lehn et al., a larger portion (if not all) of ligand charged groups are able to position themselves at the surface of the bilayer, while the hydrophobic residues are only exposed to the bilayer center and interact favorably with lipid molecules. This arrangement leads to a substantially favorable free energy difference for the transport of the NP from the water phase to the bilayer core. For NPs of other sizes this may not be the case, resulting in less negative, zero and even positive free energy differences, in agreement with the observations here. We also emphasize that the process of a NP exiting the membrane may involve a different pathway from the NP entering the membrane. For example, as has been recently observed in our own studies, while a “naked” NP particle may attach to the membrane followed by fusion with it, NP exit from the membrane can occur only in association with lipid molecules attached to it [15]. This invariably complicates the definition of a consistent reaction coordinate and thus PMF calculation. In the present study, we only consider the process of a pristine NP entering (or fusing with) the membrane from the bulk water phase.

### The presence of NP induces local disorder and cholesterol depletion in the lipid bilayer

We further examined the re-organization of the lipid bilayer structure upon NP inclusion in the unbiased MD simulations, where the NP is initially placed in the bilayer core. Lipid bilayers composed of a single phospholipid species undergo a well-defined phase transition in which the lipid chains change from an ordered or gel state (Lg) to a fluid or liquid-disordered (Ld) state. Within the physiologically relevant temperature range (30–40°C), addition of cholesterol in a concentration above 30% mol. eliminates the Lg-Ld phase transition, and a new, distinct state of the bilayer is observed, termed the liquid-ordered (Lo) phase [32], [33]. This new phase is characterized by an intermediate fluidity between those of the gel and the fluid phases [34].

**Figure 4:**
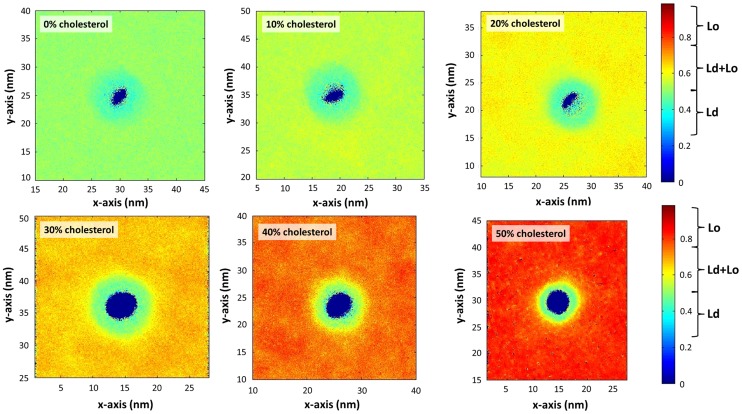
Spatially averaged lipid order parameters for bilayer systems with different cholesterol concentrations. The characteristic values P_2_ order parameter for various bilayer phases are demarcated in the color bar on the right [35]. The different box sizes are due to the gradual condensing effect of cholesterol on the bilayer.

To describe the structure of the lipid bilayer in the vicinity of the NP, we calculated the spatially averaged second-rank lipid order parameter, 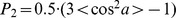, which is a metric of the order of the lipid hydrocarbon chains and characterizes the alignment of lipid molecules with the bilayer normal (Figure 4). The square brackets denote an ensemble average and *α* is the angle between the bond formed by two CG beads and the bilayer normal. A value of 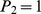 corresponds to perfect alignment with the bilayer normal, 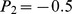 to perfect anti-alignment, and 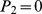 to a random orientation of molecules with respect to the bilayer normal. The Ld phase is characterized by order parameters ranging between 0.2–0.5 and the Lo phase order parameters are in the range 0.7–1 [35] (see also the color bar scale of Figure 4). In Figure 4 it is shown that the presence of the NP induces a local increase in the disorder of lipids in all systems. Remarkably, for the systems with high cholesterol concentration (more than 30% mol.), we find that in a spherical segment area of approximately 2–3 nm (depending on cholesterol concentration) radius around the NP, the bilayer is in the liquid-disordered (Ld) phase for all the systems as indicated by the average lipid tail order parameter of ∼0.4.

**Figure 5:**
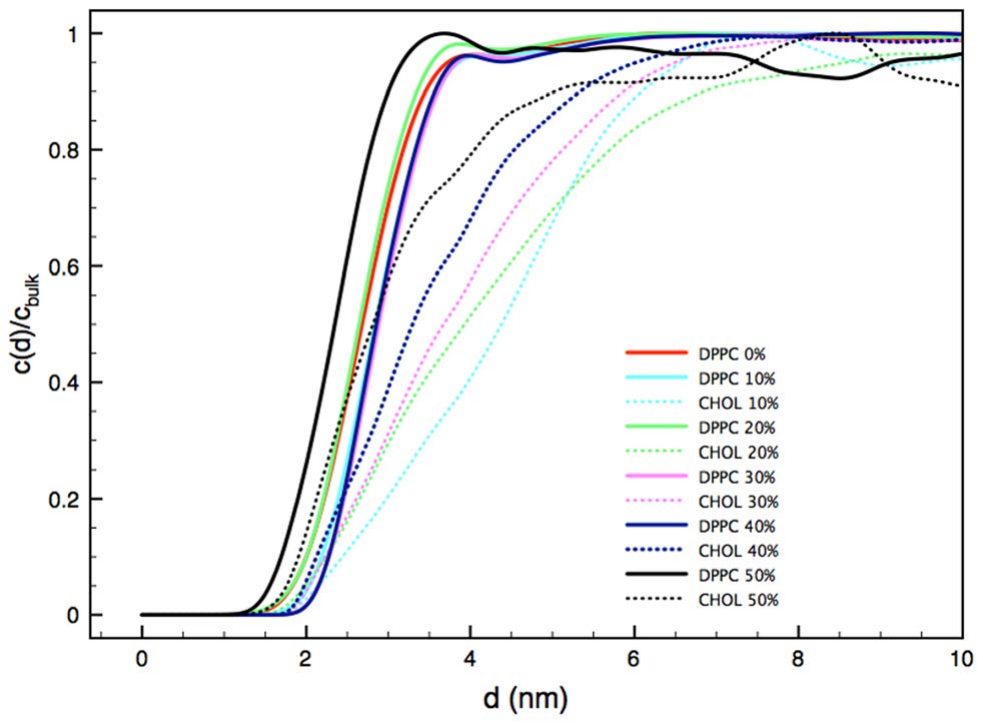
2D Radial concentration of DPPC and CHOL from the NP center of mass. The concentration values as a function of the distance from the NP center of mass, c(d), are normalized with respect to the bulk values, c_bulk_.

To further characterize the local structure of the lipid bilayer in the vicinity of the NP, we focused on two additional structural metrics. Radial concentration profiles, *c(d)*, describe the concentration of various molecular species as a function of distance from the NP center of mass. Plotted in Figure 5, these profiles are normalized with the bulk concentration, 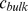, of respective species. In addition, Radial Distribution Functions (RDFs) between the MUS terminal groups on the NP surface and various groups on DPPC and cholesterol molecules are shown in Figure S9 in Text S1. The radial concentration profiles of DPPC molecules do not exhibit a significant variation across different systems (Figure 5). However, the behavior of the cholesterol radial concentration profiles is different from that of DPPC groups in several aspects. According to these profiles, the normalized cholesterol concentration is lower in the vicinity of the NP, compared to DPPC concentration, and the effect of the NP presence on cholesterol distribution is still evident even beyond 6 nm away from the NP centre. Furthermore, these radial concentration profiles exhibit substantial variation across different systems, compared to the analogous DPPC profiles. The RDF analysis supports this picture. As seen in Figure S9 in Text S1, cholesterol density is depleted in the presence of the NP, compared to the bulk value, and these effects are seen up to and, depending on the concentration, beyond a 6-nm separation distance from MUS groups. In addition, Table S2 in Text S1 shows the concentration of cholesterol in the 3 nm vicinity of the negatively charged MUS terminal group in comparison with the bulk concentration. This data provides further evidence that the NP prefers to interact with DPPC molecules rather than cholesterol, leading to a local depletion of cholesterol concentration in the vicinity of NP and formation of a region around NP with characteristic features of the liquid-disordered (Ld) phase.

**Figure 6:**
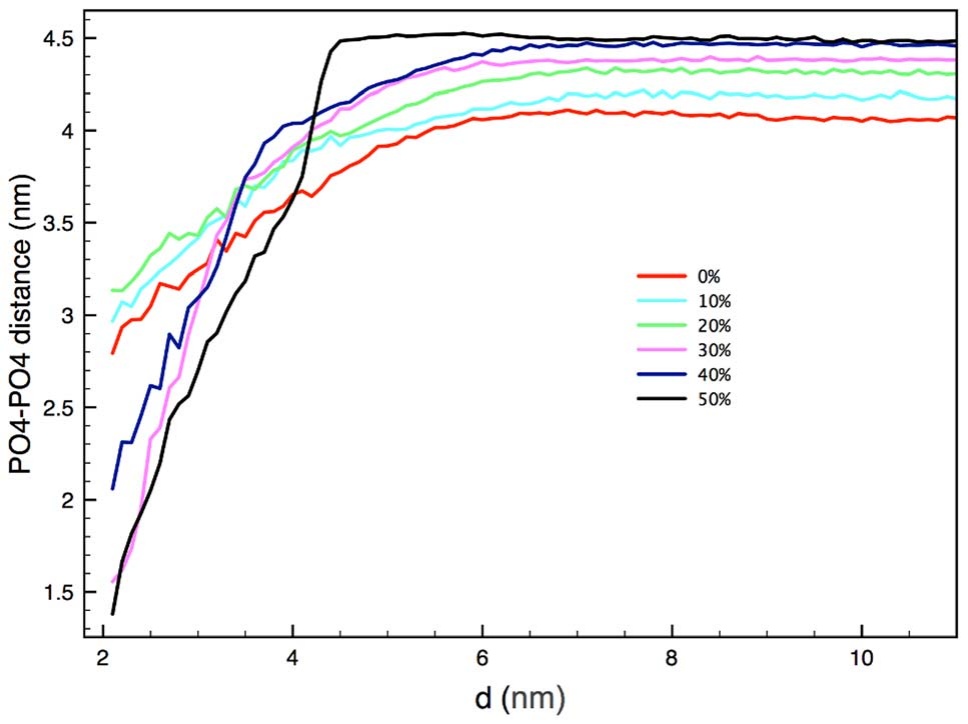
Bilayer thickness, defined as the distance between phosphate groups (PO4) of different bilayer leaflets, for different cholesterol concentrations as a function of the distance from the NP-center of mass.

To quantify the preference between the NP and the DPPC/cholesterol molecules, we calculated interaction energies between different species in the system (per NP). In Table 1 interaction energies normalized with respect to the number of molecules within the cut-off distance of 1.2 nm, are shown. At low concentrations of cholesterol, cholesterol molecules tend to move away from the NP, leading to lower interaction energies. As the concentration increases, crowding effects force cholesterol molecules to be closer to the NP, leading to higher values of interaction energies per molecule. This behavior is consistent with the concentration as a function of the distance from the NP center, *c(d)*, and the 2D RDF analysis presented in Figures 5, 6, and Figure S6 in Text S1. In Figure 6 is shown the radial bilayer thickness relative to the NP center (Figure S10 in Text S1 provides an additional metric and analysis of the bilayer thickness). Close to the NP (i.e. at distances of up to 3–4 nm), a thinning of the membrane is observed with respect to the bulk thickness of the bilayer (distances over 4 nm, where the bilayer thickness reaches a plateau value). The strongest effect on the bilayer thickness is observed in the 50% mol. cholesterol bilayer case (Figure 6 and Figure S10 in Text S1). This bilayer thinning can be correlated to the increase of lipid disorder in the vicinity of the NP due to cholesterol depletion; lipid disordering is known to lead to reduced membrane thickness [34]. Moreover, the positively charged phosphate groups of lipid heads are attracted by the negatively charged MUS ligand ends, and are dragged towards the inner part of the bilayer (Figure S11 in Text S1). This effect contributed to the local thinning of the membrane caused by cholesterol depletion and increase in lipid disorder.

**Table 1:** Normalized interaction energies of the NP with the different components of the system in KJ/mol.

Chol.	DPPC head	DPPC tail	Chol
0%	−0.94±0.01	−4.45±0.01	N/A
10%	−0.97±0.01	−4.91±0.02	−1.60±0.35
20%	−0.94±0.01	−4.59±0.02	−1.73±0.21
30%	−1.06±0.01	−5.20±0.05	−2.83±0.26
40%	−1.10±0.01	−4.99±0.05	−3.21±0.14
50%	−0.82±0.01	−3.87±0.05	−2.96±0.12

DPPC molecules interact preferentially with the NP. The normalization was done with respect to the number of molecules within the cut off distance of 1.2 nm.

## Discussion

The partition mechanism of a striped anionic NP in lipid bilayers is studied herein using state-of-the-art biased and unbiased MD simulations. At the beginning of the translocation process, when the NP is on the surface of the bilayer, the ligand-decorated, “hairy” nature of the NP hinders the self-assembly and aggregation of lipids on the surface of the NP obstructing it from entering the bilayer. As a result, in contrast to previous studies of smooth NPs with similar size and polarity [9], [15], unbiased MD simulations did not capture the actual process of NP fusion and translocation through the membrane on the 10 µs timescale. We note here that recently insights into why such events may require even longer simulation times to be directly detected in molecular dynamics have emerged [36].

NP insertion in the bilayer, studied here by biased and unbiased MD simulations, causes the NP surface ligands to rearrange and “snorkel” the charged groups towards the lipid head-groups, while at the same time the ligand hydrophobic chains remain buried inside the bilayer core. This ligand re-arrangement also leads to formation of contacts and more enhanced interactions between DPPC molecules and hydrophobic ligands on the surface of the NP. Although spontaneous insertion of a NP into the bilayer is not observed in equilibrium MD simulations here, we note that the free energy barriers observed for the NP are much lower than that for the translocation of a single ion. We conclude, that it is this snorkeling mechanism which is responsible for these relatively low free energy barriers. Comparison of these observations with the results of van Lehn et al. [24] suggests, however, that the snorkeling mechanism and the resulting free energy profiles should strongly depend on ligand flexibility and length, as well as on the model of the lipid bilayer. At the same time, this variation in the behavior also implies new opportunities for the design of NPs, based on the structure of ligands as the tuning parameter.

The entrapment of the NP in the bilayer core leads to an energy penalty and positive free energy barrier. Depending on the NP size or effective size, the results presented by van Lehn et al. start from small negative values of the free energy difference, go through deep negative minima and then reach small positive values. In some cases reported in Ref. 24, a large portion (or even all) of ligand charged groups are able to position themselves at the surface of the bilayer, while the hydrophobic residues are only exposed to the bilayer center and interact favorably with lipid molecules due to simulation methodology used. This arrangement leads to a substantially favorable free energy difference of certain NPs between the water phase to the bilayer core. For NPs of other sizes this is not the case, resulting in less negative, zero and even positive free energy differences, in agreement with the observations here.

While the snorkeling of the charged groups towards the surface of the lipid bilayer could be an important mechanism of transport facilitation, it may be hampered by the presence of cholesterol that rigidifies and orders the membrane. To study the effect of cholesterol concentration in the NP translocation, six different bilayers with cholesterol concentrations ranging from 0% to 50% mol. are investigated with unbiased MD simulations. We observe that flexible ligands on the NP surface induce formation of a region corresponding to liquid-disordered phase in the vicinity of the NP in all studied systems. This phenomenon is due to the NP preferential interaction with the DPPC lipids, which leads to effective expulsion of cholesterol around the NP. As a result of the existence of cholesterol lean area around the NP, we could not observe any definite impact of the cholesterol presence on the ability of ligands to snorkel (see Figure S12 in Text S1). This however requires further investigation.

In the absence of unbiased MD simulations capturing the actual process of fusion and translocation, here we attempt to infer possible scenarios prompted by the relative mobility of the membrane components. The DPPC lipid and cholesterol self-diffusion coefficients were calculated using the unbiased MD simulations. The Mean Square Displacement (MSD) and the technical details of the diffusion coefficient calculations are presented in section F of Text S1 (Figure S13 in Text S1 and Table S3 in Text S1). At 40% mol. cholesterol, the cholesterol self-diffusion coefficient is 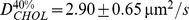, same as the one for cholesterol (see also Table S3 in Text S1). In contrast, at 10% mol. cholesterol, the cholesterol self-diffusion coefficient is 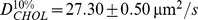, significantly higher than that for the 40% case. Hence, the mobility of cholesterol strongly depends on the cholesterol concentration of the membrane and thus, in cholesterol-rich membranes, the local re-arrangement of the membrane is possibly hindered by slow diffusion of cholesterol molecules, and the whole fusion and translocation process may become diffusion-limited.

These preliminary considerations implicate yet another possible scenario. A situation is possible where several NPs are already incorporated in the membrane form and stabilize between them larger patches of cholesterol-lean membrane. These patches may feature much higher permeation rates, compared to other regions of the membrane, due to greater mobility of molecules in these patches, higher disorder and lower thickness of the membrane. Such a co-operative adsorption mechanism in the presence of multiple NPs could explain the nonlinear cellular uptake with concentration as has been recently observed [6].

This strong dependence of structural reorganization of the membranes on the presence and concentration of cholesterol significantly affects the drug delivery process in various contexts. With certain cancer cells containing higher levels of cholesterol, it is clear that design of efficient NPs for cancer therapy must be based on a better understanding of the impact of cholesterol on the nanomaterial cellular uptake mechanisms. Therefore, strategies for tailored decoration of NPs aiming to selectively target specific cells based on their cholesterol content, are required. This study is an early step in this direction; moreover, it highlights the importance of a realistic representation of the system under investigation and long simulation timescales, required to capture NP translocation in its full complexity.

## Methods

### Coarse-grained model

In the present study, all membranes are described as two-component lipid bilayers using the MARTINI CG model for lipids, cholesterol, and water [19]. The MARTINI model was also employed for the construction of the NP using an approach previously introduced by us [15]. We emphasize that the NP under investigation, although inspired by previous studies, is purely representative, and its dimension, pattern, and charge are exactly not those of any specific experimentally studied nanoparticle. The CG simulations presented in this article were performed with the GROMACS simulation package, version 4.5.5 [37].

The lipid bilayers were constructed using ∼4,000–8,000 1,2-dipalmitoyl-sn-glycero-3-phosphocholine (DPPC) lipid molecules and the respective cholesterol molecules to achieve the desired cholesterol concentrations. To setup each system we performed the following procedure: (a) mixing system components starting from random initial positioning of lipids, cholesterol, and water. The mixture was simulated for several tens of nanoseconds until the eventual formation of the bilayer was observed, (b) several properties of the bilayer were then checked in order to ensure almost perfectly symmetric distribution to within 10 molecules of cholesterol and lipids among the two leaflets since an asymmetric cholesterol distribution affects membrane curvature [38], and (c) in addition, occasional runs with larger simulation boxes were performed to assess finite size effects. In all instances, bilayers were simulated in excess water, with approximately 100 water molecules per lipid (or, equivalently, 28 CG water molecules per lipid), a ratio well above the degree of hydration observed in multilamellar vesicles for fluid (Ld) bilayers and the even less hydrated gel (Lg) bilayers [39]. The water phase has been almost doubled from the original lipid∶CG water ratio of 1∶15 suggested by Ref. 7 to allow for the complete inclusion of the NP and in order to ensure that individual uncoupled bilayers separated by bulk water will be simulated, thus avoiding artifacts caused by interacting periodic images of the system.

Each system was initially equilibrated for 1 µs. Within this time, tensionless membranes were formed and thermodynamic properties converged to equilibrium values. During the simulation course the lipids remain in the bilayer and do not break in cylindrical micelles. Following equilibration, an NP was placed at the center of the bilayer using VMD [40].

### Simulation parameters

MD simulations were performed with constant pressure, temperature, and number of particles (NPT ensemble) [37]. The temperature was kept constant at 323 K using the Berendsen thermostat with a relaxation time of 1.25 ps [41]. The pressure of the system was semi-isotropically coupled and maintained at 1 bar using the Berendsen algorithm with a time constant of 0.22 ps and a compressibility of 3e-5bar^−1^. The non-bonded potential energy functions were cut off and shifted at 12 Å, smoothly decaying between 9 and 12 Å for van der Waals forces and throughout the whole interaction range for the treatment of electrostatic forces. The simulations were performed using a 20 fs integration time step. Six different cholesterol ratios, ranging from a cholesterol-free bilayer to a 50% ratio were considered; the exact composition of the different membranes is presented in Table S1 in Text S1.

### Potential of mean force calculations

The Potential of Mean Force (PMF) as a function of the distance between the NP and the center of the lipid bilayer has been calculated using the Umbrella Sampling protocol of Gromacs for the cholesterol-free membrane and the membrane containing 50% mol. cholesterol concentration. The distance between the initial NP position and the center of the bilayer was 7 nm, which was divided into 36 windows of 0.2 nm each. In each window, a different initial configuration was set up with the NP placed at the corresponding distance from the center of the bilayer. Then, the system was subjected to four subsequent annealing simulations at 450 K, 400 K, 380 K, and finally 323 K. The temperature coupling was restarted at the 0 ps value after 400 ps, 1000 ps, and 2000 ps. Then, the biasing potential was applied in order to restrain the NP at the given position, and the system was left to equilibrate at the restrained position for 200 ns. This procedure was followed in order for the lipids to re-arrange correctly around the NP. Subsequently, the system was simulated for another 400 ns in the case of 0% mol. cholesterol and 300 ns in the case of 50% mol. cholesterol, with the biasing potential applied to restrain the center of mass of the NP at a required distance from the center of the bilayer. A single PMF profile required 36 simulations (36 windows) and a total simulation time of 21.6 µs (18 µs in the 50% mol. cholesterol membrane). A force constant of 750 kJ mol^−1^ nm^−2^ was applied, following the approach by Gkeka et al. [15] To examine the PMF sampling and convergence in further detail, we investigated the PMF change when increasing the sampling time for each window starting with 50 ns up to 400 ns (or 300 ns for the cholesterol-containing membrane) with 50 ns intervals. The initial equilibration period was 200 ns in all cases.

The simulated system features a large enough water phase to avoid possible effects associated with the system size and NP-NP interactions over periodic boundaries, as tested by considering both larger and smaller systems. Note that for the 50% cholesterol system, we doubled the size of system in order to avoid any tension coupling between the periodic image of the NP, so the final system contained ≈14,000 lipids and ≈400,000 CG water particles (∼2,000,000 water molecules) corresponding to a system size of 648,302 CG particles. This system size was used to avoid finite size effects and artifacts from the nano-object interacting with its periodic images. In Figure S14 in Text S1 we present a representative simulation snapshot at zero NP-bilayer distance, which demonstrates that despite the size of the membrane, minimal buckling was involved. The NP was left free to rotate around its restrained center of mass. In order to obtain the unbiased PMFs, we used the weighted histogram analysis method (WHAM) [42] with 200 bins and a tolerance of 10^−7^ for the convergence of WHAM equations.

## Supporting Information

Text S1 Supplementary information includes description of the employed model, force field, the simulation protocol, and further illustrative plots.
(DOCX)

Dataset S1 The data used to generate Figures 2, 3, 4, 5, and 6.
(ZIP)
