# Correction: Membrane Partitioning of Anionic, Ligand-Coated Nanoparticles Is Accompanied by Ligand Snorkeling, Local Disordering, and Cholesterol Depletion

**DOI:** 10.1371/journal.pcbi.1004769

**Published:** 2016-02-17

**Authors:** The PLOS Computational Biology Staff

There are some typographical errors in the manuscript. In the Results section of the manuscript, specifically in the paragraph entitled “Partitioning mechanism of a striped anionic NP in lipid bilayers”, it is stated that “We consider an anionic NP with a core diameter of 4.3 nm…”. The correct statement should be “We consider an anionic NP with a core diameter of 3 nm…”

There is also an error in S1 Fig: the distance between the C1 and the Qa particle is not 0.62 nm as shown in the figure, but 0.47 nm. Please view the correct version of S1 Fig here:

**S1 Fig:**
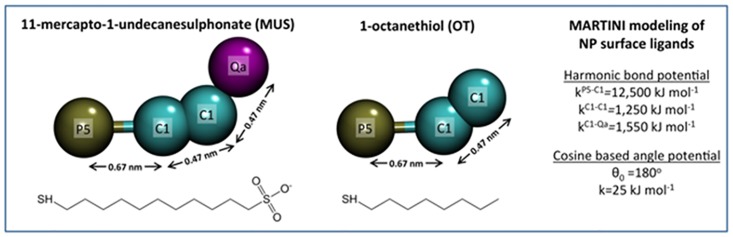
Coarse-grained models of the NP surface ligands used in the present study. Colors: Negative beads bearing -1e charge (Qa) = purple; hydrophobic beads (C1) = cyan; and polar beads (P5) = ochre. (TIF)
