# Supplementary material for: Membrane Partitioning of Anionic, Ligand-Coated Nanoparticles Is Accompanied by Ligand Snorkeling, Local Disordering, and Cholesterol Depletion
Source: PLoS Comput Biol. 2014 Dec 4;10(12):e1003917. doi: 10.1371/journal.pcbi.1003917 (PMC4256007; doi:10.1371/journal.pcbi.1003917)
Supplement: Text S1 — Supplementary information includes description of the employed model, force field, the simulation protocol, and further illustrative plots. (DOCX) [file pcbi.1003917.s001.docx]

**Supporting Information**

Membrane partitioning of anionic, ligand-coated nanoparticles is accompanied by ligand snorkeling, local disordering, and cholesterol depletion

**Paraskevi Gkeka,∗,1 Panagiotis Angelikopoulos,2 Lev Sarkisov,3 and Zoe Cournia∗,1**

1*Biomedical Research Foundation of the Academy of Athens, 4 Soranou Ephessiou, 11527 Athens, Greece, Computational Science and Engineering Laboratory,*

2 *Computational Science and Engineering Lab, Institute of Computational Science, D-MAVT, Clausiusstrasse 33, ETH Zurich, CH-8092, Switzerland, and*

3 *Institute for Materials and Processes, School of Engineering, The University of Edinburgh, Edinburgh, United Kingdom*

*Corresponding authors

Tel: +302106597195

Fax: +302106597545

E-mail: [pgkeka@bioacademy.gr](mailto:pgkeka@bioacademy.gr), [zcournia@bioacademy.gr](mailto:zcournia@bioacademy.gr)

**A. Systems under investigation – System setup**

The compositions and sizes of the simulated systems are shown in Table S1. In the preliminary initial setup of the biased simulations, where the membranes had the same size as in the unbiased simulations, system artifacts, such as deformation of the membrane, were observed during the equilibration period due to the force imposed on the nanoparticle (NP). These artifacts led to substantial changes in the size of the simulation box. Thus, extended systems were used to avoid interaction of the NP and/or the membrane with their periodic images in our final implementation of the biased simulations. The MARTINI CG water has a freezing temperature, which is too high compared to real water (280 K to 300 K depending on the simulation conditions). Also, apart from simulations performed at low temperatures, rapid freezing has been observed in systems where a nucleation site is already present (a solid surface, or like in our case a bilayer surface). [1,2] Thus, in order to avoid this unphysical freezing of the CG water, we added 10% mol. of antifreeze water particles to ensure that the water present in our systems is in the liquid phase. Antifreeze CG water is a special type of CG particle within the MARTINI model, which is commonly used to avoid freezing artifacts arising from the GC force field. [1,3]

**Table S1.** Systems under investigation.

| **Cholesterol concentration** | **# of cholesterol molecules** | **# DPPC lipids** | **# CG water** | **# Na+** |
| --- | --- | --- | --- | --- |
| 0% (unbiased MD) | 0 | 8,124 | 234,423 | 134 |
| 0% (biased MD) | 0 | 7,946 | 297,065 | 134 |
| 10% | 799 | 7,179 | 167,577 | 134 |
| 20% | 1,504 | 6,014 | 175,429 | 134 |
| 30% | 2,241 | 5,237 | 157,887 | 134 |
| 40% | 4,017 | 6,022 | 151,324 | 134 |
| 50% | 3,741 | 3,735^[[1]](#footnote-1)^ | 176,227 | 134 |

A detailed description of the NP surface ligands in a Coarse-Grained (CG) representation is shown in Figure S1. The first long bond between the P5 bead of the NP core and C1 bead of the ligand is designed to represent the Au-S bond of the thiol group, without explicitly representing the thiol group as a CG bead. [4] The construction of the NP is described in detail used in our previous studies [5] and is inspired by the work of Verma et al., [6] where a possible striped pattern of ligands on the surface of the NP is suggested. We note that our CG NP does not replicate exactly the NP developed by Stellaci and co-workers, [7] but is rather a qualitative interpretation of its key characteristics.


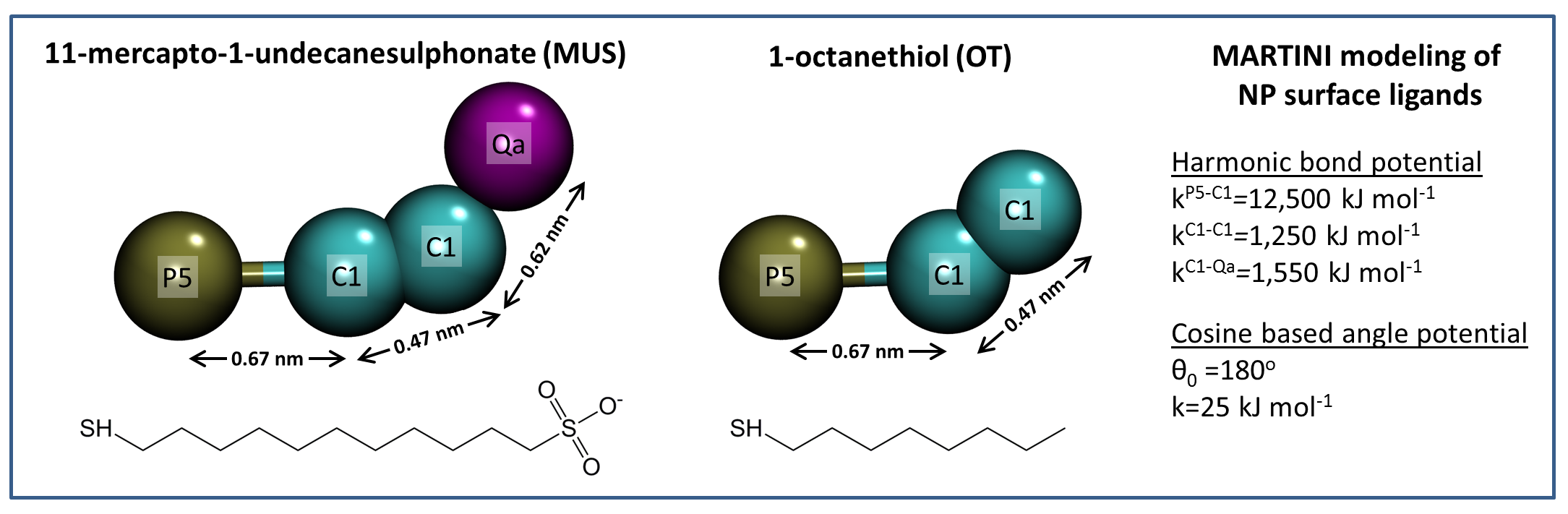


**Figure S1.** Coarse-grained models of the NP surface ligands used in the present study. Colors: Negative beads bearing -1e charge (Qa) = purple; hydrophobic beads (C1) = cyan; and polar beads (P5) = ochre.

## B. Mechanism of partitioning of striped anionic NPs in lipid bilayers

We studied the translocation of a sodium ion through a DPPC membrane comprising 0% and 50% mol. cholesterol by Potential of Mean Force (PMF) calculations. To construct the PMF, umbrella sampling was used and the ion was restrained at different distances from the center of the bilayer. A total of 30 Umbrella sampling windows were used, with a restraining force constant of 500 kJ mol^-1^ nm^-2^. Each window was simulated for 25 ns. All the simulations were performed using the MARTINI force field as applied in Gromacs 4.5.5. The PMFs for the two different systems are shown in Figure S2.

**
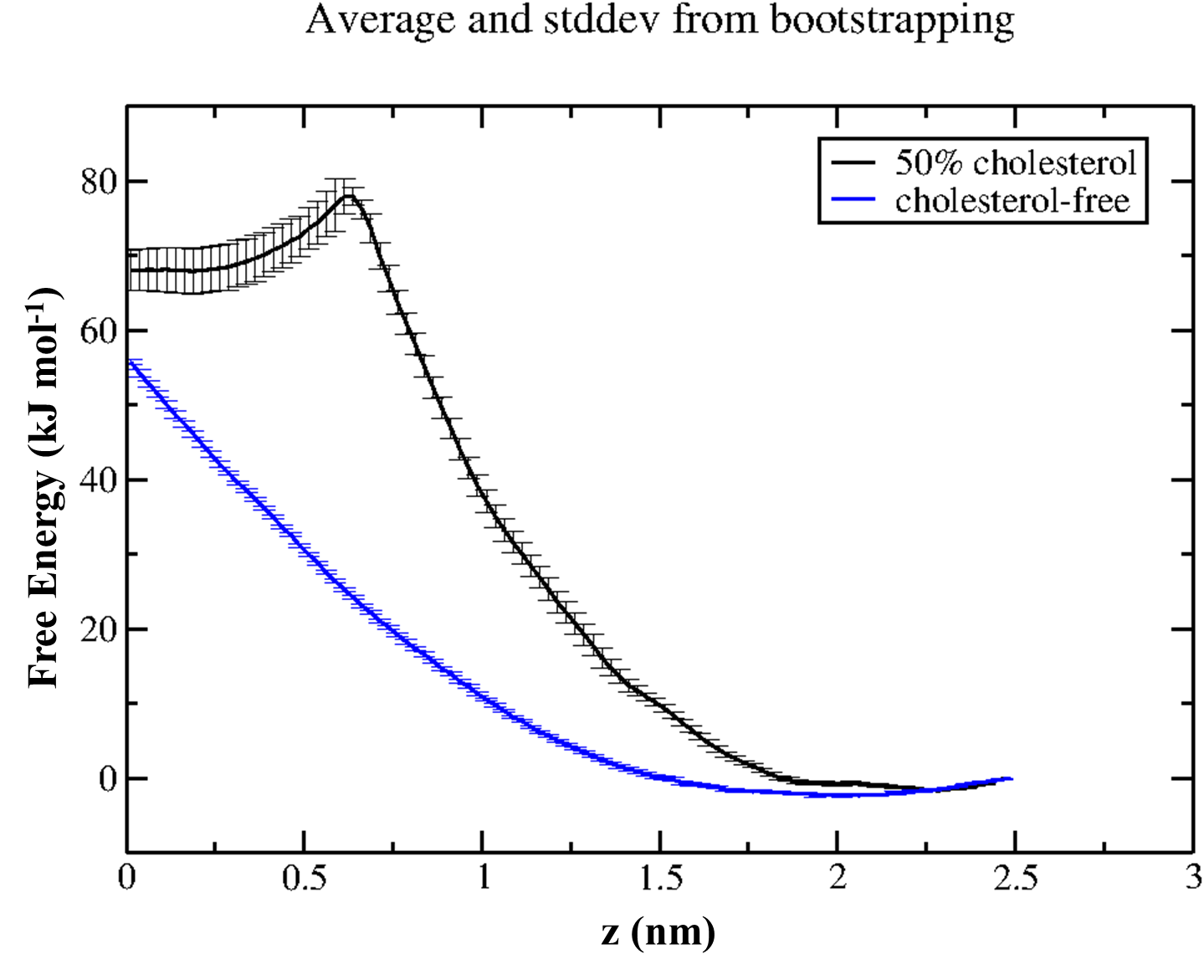
**

**Figure S2.** PMFs of Na+ translocation through a DPPC bilayer with 0% (blue line) and 50% (black line) mol. cholesterol, with respect to the distance from the membrane center. z is the distance from the bilayer midpoint in nm. The errors were calculated using the bootstrap method.

Following the PMF calculations of the Na^+^ translocation across the two bilayers (0% and 50% mol. cholesterol), unbiased simulations placing the NP in the water phase 4 nm away from the bilayer surface were performed. The total simulation time was 10 µs for all systems except the system with 50% mol. cholesterol, where the simulation was performed for 8.5 µs. In all cases, the NP partitioned at the surface of the bilayer within the first 50 ns of each simulation (Figure S3). To examine potential rearrangements of the NP ligands upon partitioning into the bilayer, we calculated the number density maps of the charged ligand ends (Figure S4). The number density was calculated using the VolMap plugin in VMD, which creates volumetric maps (3D grids containing a value at each grid point) based on the atomic coordinates and properties of a specified atom selection. Each grid point is set to either 0 or 1, depending on whether it contains no atoms (0) or one or more atoms (1). This number was averaged over the last 500 ns of each simulation, providing the number density (or fractional occupancy) of that grid point. Finally, in Figure S5 we present the final snapshots from the simulations with the NP inserted in the membrane.


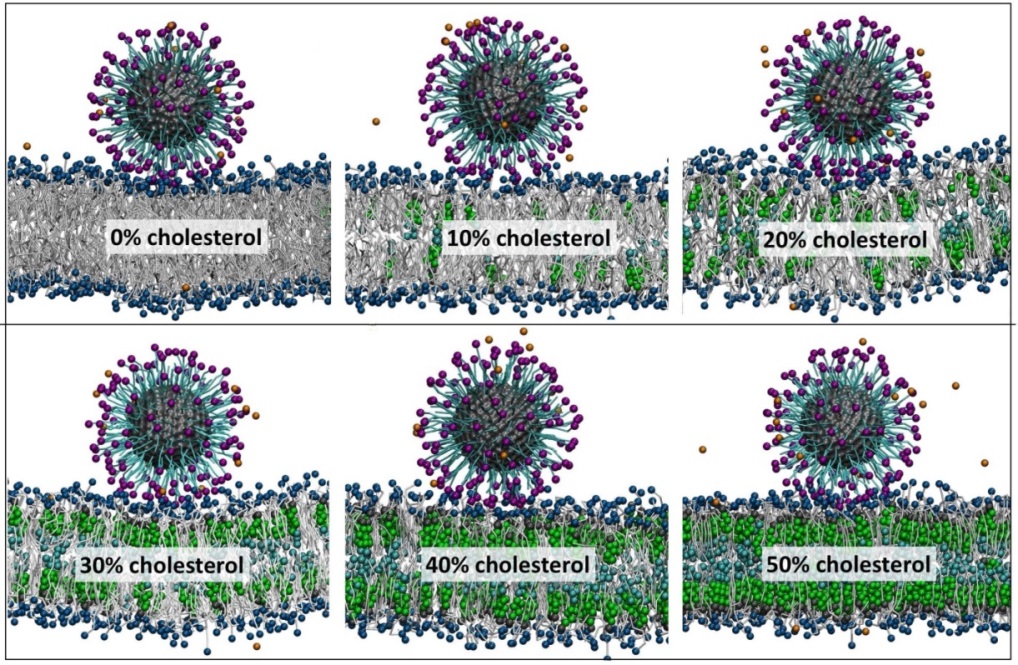


**Figure S3**. Final snapshots from the simulations with the NP initially placed in the water phase of the membrane systems. Colors: NP core=gray, negatively charged ligand end groups=purple, positively charged lipid head groups=blue, hydrophobic ligand tails=cyan, hydrophobic lipid tails=light grey, sodium ions=orange, cholesterol hydroxyl group=gray, cholesterol ring particles=green, cholesterol hydrophobic tail=cyan.

**
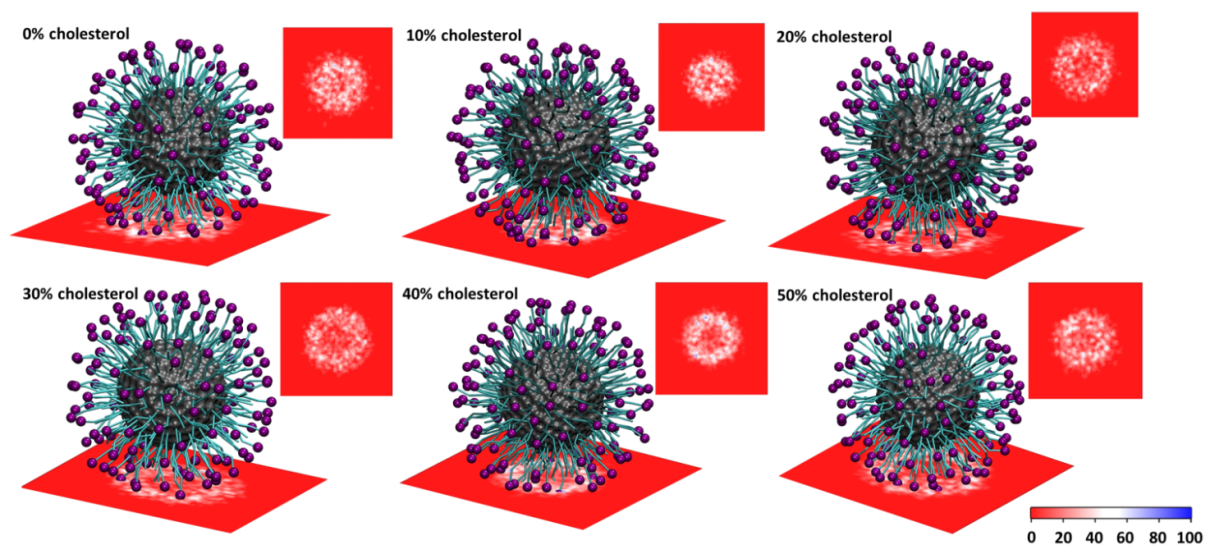
**

**Figure S4.** Number density maps of the negatively charged end-terminal groups of the NP ligands over the last 500 ns of the simulation for the different systems with the NP initially placed in water, 4nm away from the membrane surface. Each grid point is set to either 0 or 1, depending on whether it no atoms (0) or one or more atoms (1). The colormap indicates the total number of negatively charged CG particles in the specific grid cell. The coloring of the NP is the same as in Figure S3.

**
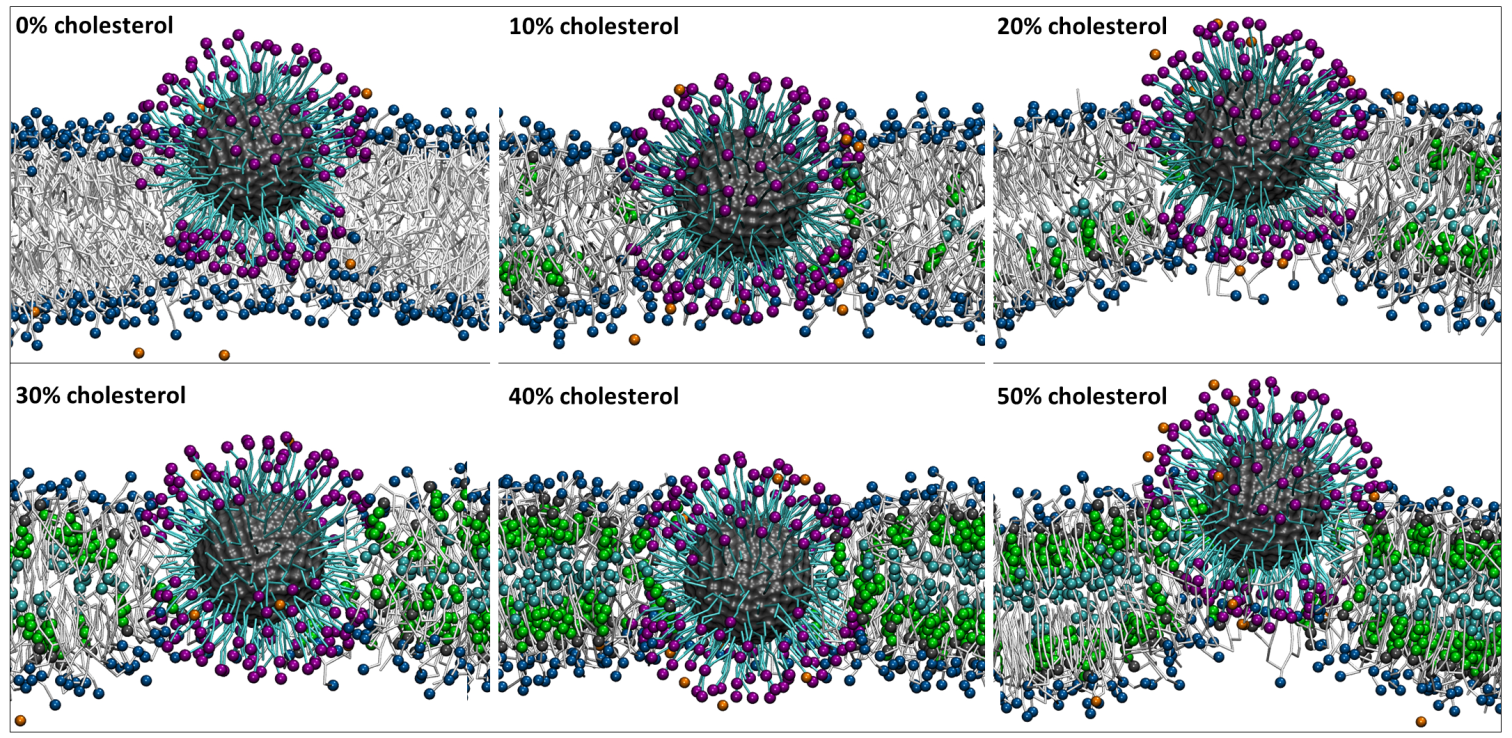
**

**Figure S5.** Final snapshots from the simulations with the NP inserted in the membrane. The coloring is the same as in Figure S3.

## C. Potential of Mean Force convergence

Below we present the results of our convergence studies of the PMF calculations. According to these results, the most significant drift of the PMF values is associated with the location of the NP in the water phase, particularly between 50ns and 100ns sampling time. As the sampling time increases, the PMF develops a more pronounced minimum at the interface of the bilayer. It is interesting to note, however, that the PMF profile between this minimum and the bilayer core changes insignificantly as the sampling time increases. As seen in Figure S7, the minimum fluctuates only between 27 kJ/mol and 29 kJ/mol between sampling times of 50 up to 400 ns. This calculation adheres to the good practices recommended by Neale et al. [8] To address the context of sampling time per window, we mention that Neale et al. employ 205 ns of simulation time for each window in the Umbrella sampling in fully atomistic simulations. Given the typical time scale conversion factor of 4 between atomistic and MARTINI CG model, our 600 ns in the CG model would translate into 2.4 µs simulation time (equilibration + sampling) per window if matched onto atomistic simulations.


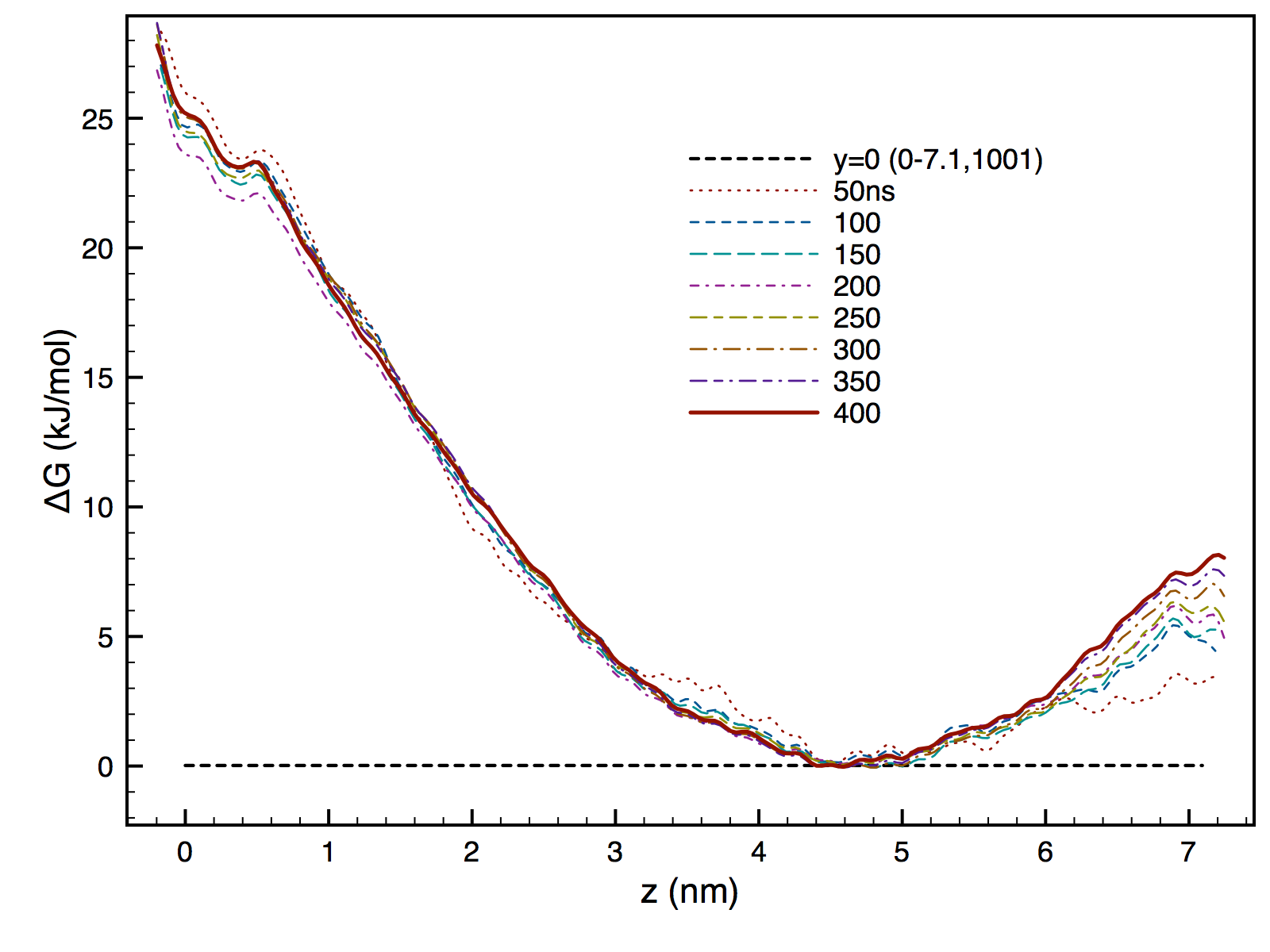


**Figure S6.** Convergence of the PMF plots with regard to additional time sampling. Legend indicates the sampling period.


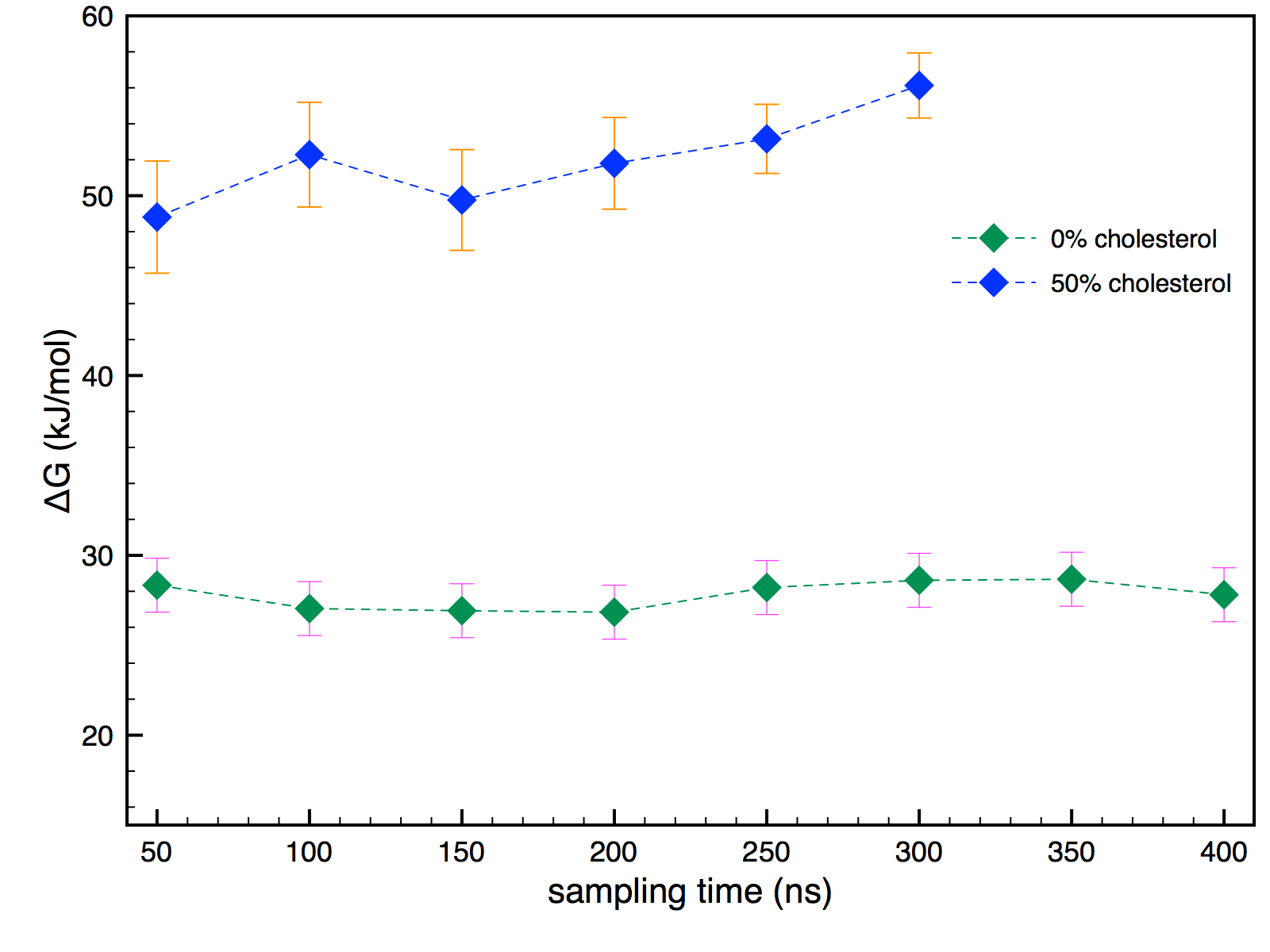


**Figure S7.** Free energy difference for the insertion of the NP from its minimum (located at the interface at z=4.5 nm for both 0% and 50% cholesterol case) to the core of the bilayer. Error bars indicate statistical uncertainty obtained via bootstrapping.

##
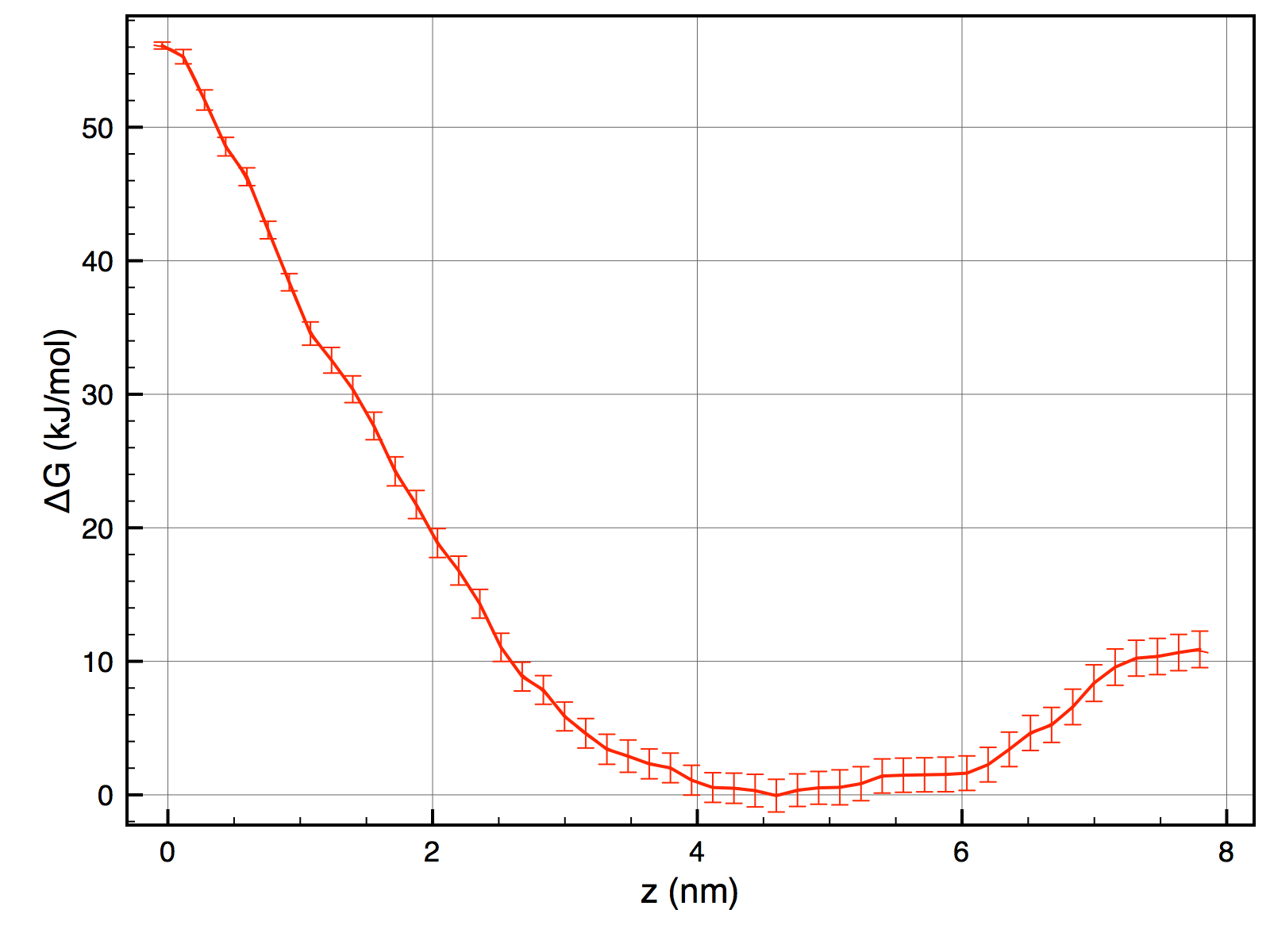


## Figure S8. PMF for NP partitioning in DPPC lipid bilayer containing 50% mol. cholesterol. The error bars represent standard deviations from two independent sets of Umbrella sampling calculations using the bootstrapping technique.

## D. The presence of NP induces local depletion in cholesterol concentration and membrane thinning

## Below we present the effect of the presence of the NP in the membranes as evidenced by local cholesterol concentration (Table S2), 2D RDF of membrane components (Figure S9), and bilayer thickness (Figure S10).

**Table S2**. Comparison of local concentration of cholesterol within 3 nm of the negatively charged MUS group and bulk concentration, as obtained from integration of the cumulative RDF. Concentrations are in % mol.

| **Bulk** | **Local** |
| --- | --- |
| 0% | 0% |
| 10% | 4% |
| 20% | 12% |
| 30% | 21% |
| 40% | 33% |
| 50% | 43% |


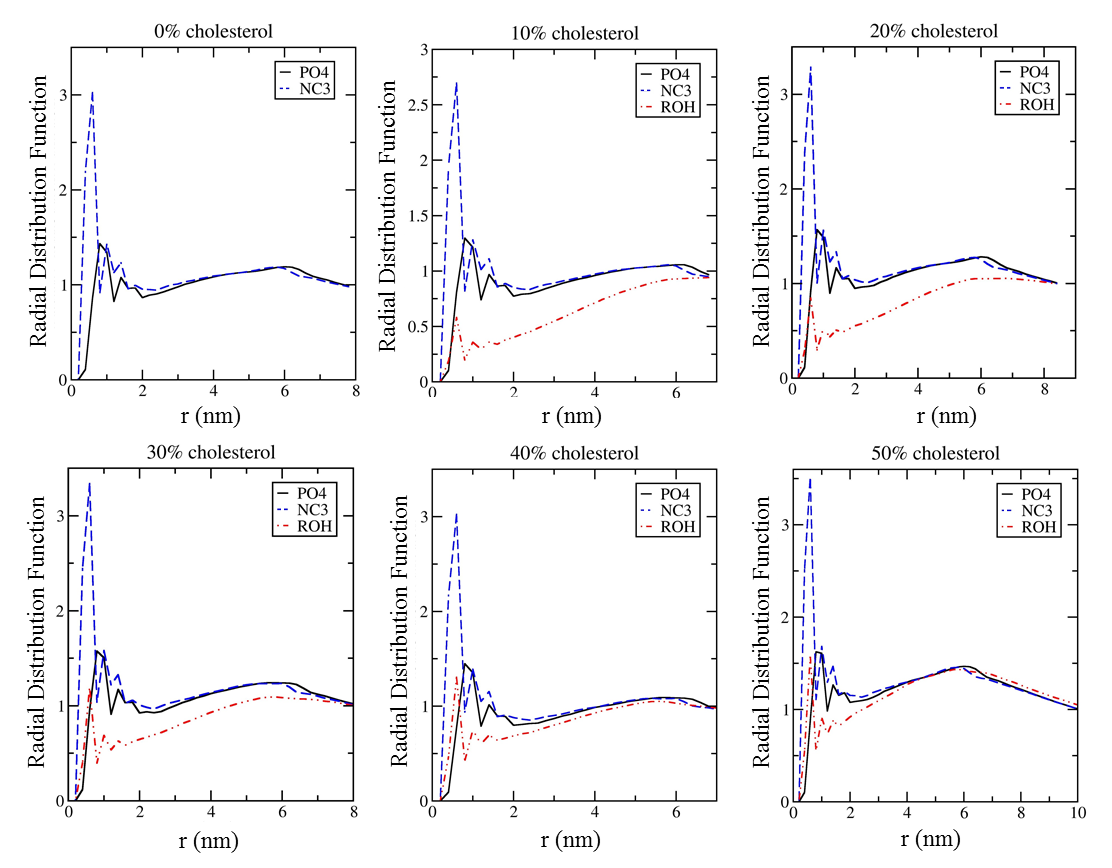


**Figure S9**. 2D Radial Distribution Function for the negatively charged MUS terminal group of the ligands and PO4, NC3 groups of lipid molecules (the first two groups) and ROH group of cholesterol, respectively.


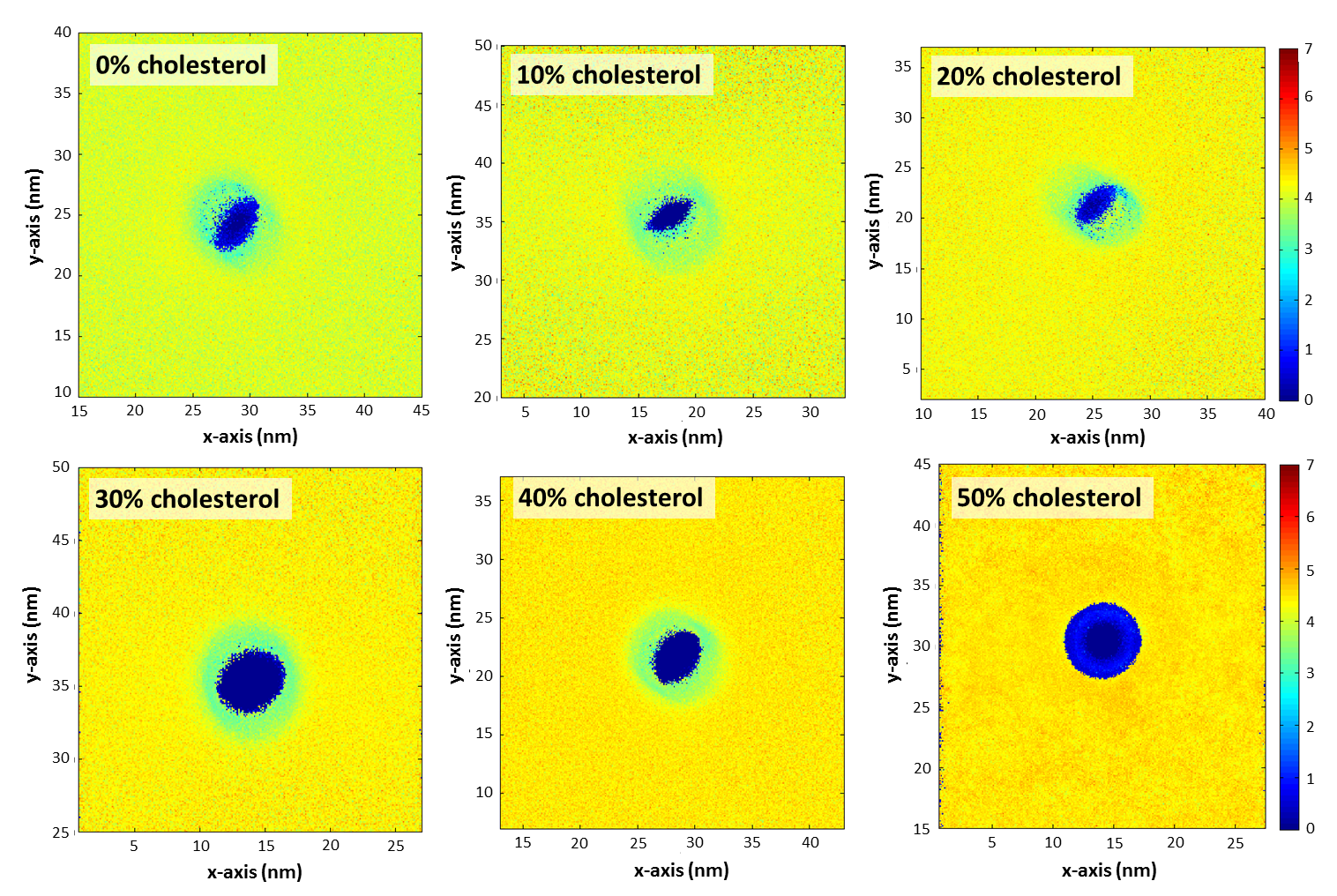


**Figure S10**. Spatially averaged bilayer thickness in nm at different cholesterol concentrations.

In Figure S11, we present the final snapshot from the simulation with the NP inserted in a membrane containing 50% mol. cholesterol. The PO4 CG particles, shown in blue VDW representation, are attracted by the negatively charged ligand ends, shown in purple VDW representation, and dragged towards the inner part of the bilayer. In addition to the thinning due to the condensation effect upon the addition of cholesterol, the positioning of the lipid head groups towards the inner part of the membrane leads to an increased local thinning of the membrane also evidenced for other cholesterol concentrations (Figure S9).


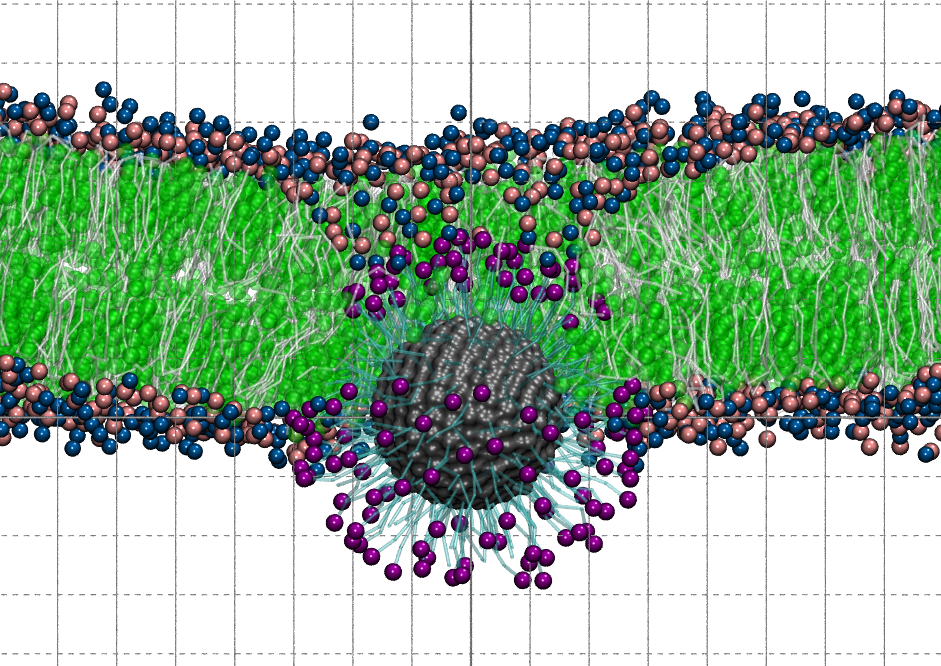


**Figure S11.** Final snapshot from the simulation with the NP inserted in a membrane containing 50% mol. cholesterol. The PO4 CG particles (blue) are dragged by the negatively charged ligand ends (purple) towards the inner part of the bilayer. The coloring is the same as in Figure S3.

**E. The effect of cholesterol concentration on the snorkeling effect**

We have calculated the density of the charged ligand termini, Qa, relative to the bilayer center (see Figure S12 below). Unfortunately, no conclusion can yet be made based on the analysis. We speculate that the reason we are unable to observe a difference in the different bilayers is that cholesterol is already depleted from the vicinity of the Qa charged ligand ends during our sampling time.


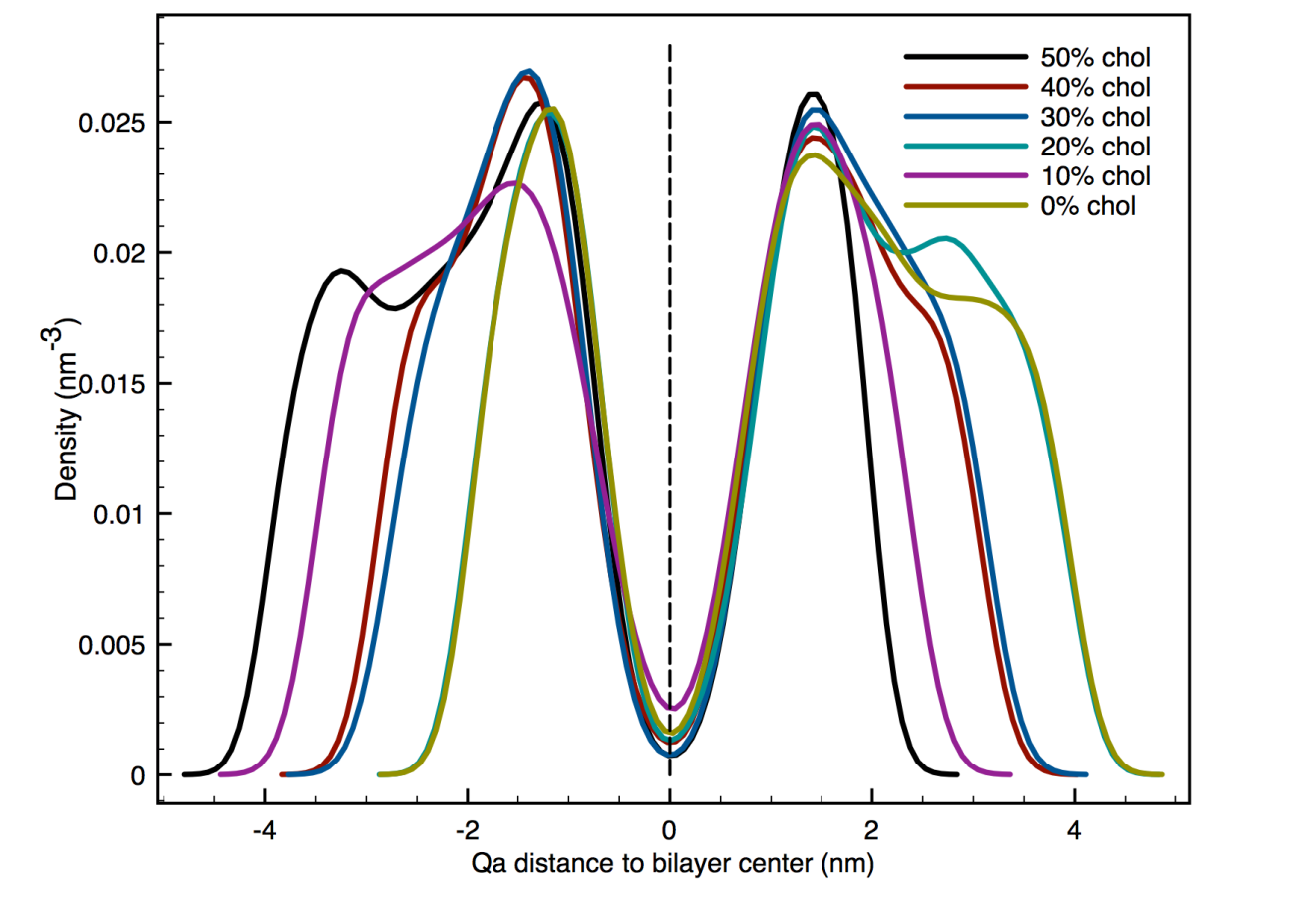


Figure S12. Density of anionic ligand ends (Qa) relative to the bilayer center.

**F. Self-diffusion coefficients**

To estimate the lateral self-diffusion coefficient of the DPPC lipids and cholesterol molecules within the lipid bilayer, we used the last 1µs of the simulations with the NP placed in water. The lateral diffusion coefficient, *D*, is related to the mean-square displacement (MSD) by

MSD = 4*D* t

where *t* is time. We can extract the lateral self-diffusion coefficient by performing linear regression of the MSD. MSD for the different systems is shown in Figure S13. Due to the different number of observations for each time interval we use the weighted least-squares method for the calculation of the slope; the times are weighted according to the number of reference points, i.e. short times have a higher weight. By using the weighted least-squares method we account for the variable uncertainty of the available data, which ensures a more accurate estimation of the error of the fit. Also in MD deviations from the linear Einstein diffusion regime are sometimes observed, particularly on short time scales. Thus, in order to exclude the initial ballistic part of MSD we start the fitting at 10% of the trajectory. Moreover, to avoid large statistical errors due to poor sampling at the larger timespans we stopped the fitting at 90% of the trajectory. The error was calculated as the difference of the diffusion coefficients obtained from fits over the two halves of the fit interval. In the case of 50% cholesterol concentration, the diffusion coefficient was calculated for the time range between 200-300ns of the last 1μs of the simulation. A conversion factor of 4 was used to divide the calculated lateral self-diffusion coefficients in order to take into account the fastest diffusion observed at the coarse-grained level due to the smoothened free energy landscape [1]. The diffusion coefficients of the cholesterol and DPPC molecules in the 50% mol. cholesterol bilayers are very low in comparison to experimental data (see for example Ref. 9). We believe that this disagreement is due to the very slow dynamics exhibited in the 50%mol. cholesterol bilayer, which cannot be captured with the specific model and in these timescales. However all the other reported self diffusion coefficients are in close agreement with experimental data [9].

**
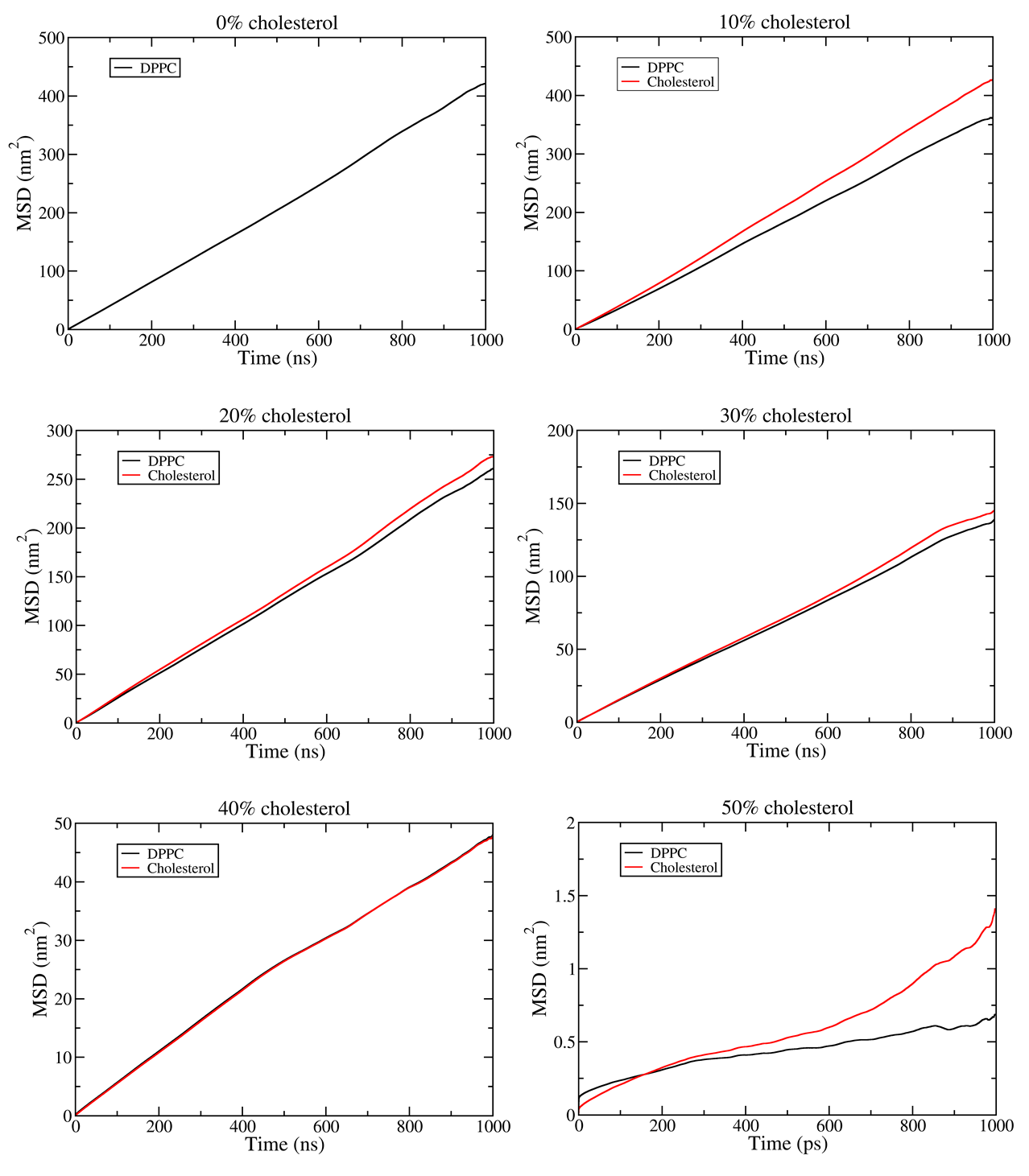
**

**Figure S13**. Mean Square Displacement of DPPC and cholesterol at different cholesterol concentrations.

**Table S3.** Lateral self-diffusion coefficients in the x-y plane for DPPC and cholesterol for the systems under investigation.

| **Cholesterol concentration** | **D [DPPC] (μm^2^/s)** | **D [CHOL] (μm^2^/s)** |
| --- | --- | --- |
| 0% | 26.68±2.63 | N/A |
| 10% | 23.43±0.08 | 27.30±0.50 |
| 20% | 16.33±1.38 | 17.08±2.00 |
| 30% | 8.73±0.80 | 9.28±1.40 |
| 40% | 2.90±0.65 | 2.90±0.65 |
| 50% | 0.05±0.03 | 0.05±0.03 |

**G. System size**

For the biased simulations a large enough membrane and corresponding water phase was used to avoid possible effects associated with the system size and NP-NP interactions over periodic boundaries, as tested by considering both larger and smaller systems. This system size was used to avoid finite size effects and artifacts from the nano-object interacting with its periodic images. In Figure S14 we show a representative simulation snapshot at zero NP-bilayer distance (window 0), which demonstrates that despite the size of the membrane, minimal buckling was involved.


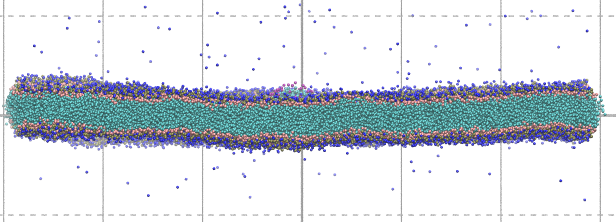


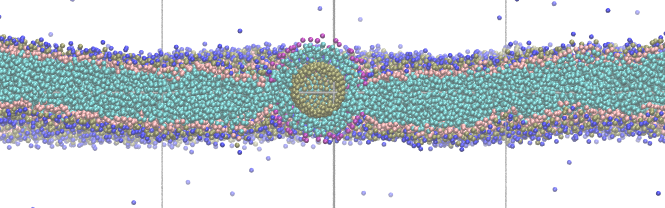


Figure S14. Top: Side view of the system at the 1^st^ PMF window excluding water. Bottom: Zoom in the bilayer that indicates the positioning the NP in the center of the bilayer. Note that there is minimal buckling involved. Each grid square demarcates a 10 nm x 10 nm area.

**H. References**

1. Marrink SJ, Risselada HJ, Yefimov S, Tieleman DP, de Vries AH (2007) The MARTINI force field: coarse grained model for biomolecular simulations. J Phys Chem B 111: 7812-7824.

2. Sangwai AV, Sureshkumar R (2011) Coarse-grained molecular dynamics simulations of the sphere to rod transition in surfactant micelles. Langmuir 27: 6628-6638.

3. Hadley KR, McCabe C (2012) Coarse-grained molecular models of water: a review. Mol Sim 38: 671-681.

4. Hakkinen H (2012) The gold-sulfur interface at the nanoscale. Nat Chem 4: 443-455.

5. Gkeka P, Sarkisov L, Angelikopoulos P (2013) Homogeneous hydrophobic-hydrophilic surface patterns enhance permeation of nanoparticles through lipid membranes. J Phys Chem Lett 4: 1907-1912.

6. Verma A, Uzun O, Hu Y, Hu Y, Han HS, et al. (2008) Surface-structure-regulated cell-membrane penetration by monolayer-protected nanoparticles. Nat Mater 7: 588-595.

7. Verma A, Stellacci F (2010) Effect of surface properties on nanoparticle-cell interactions. Small 6: 12-21.

8. Neale C, Bennett WFD, Tieleman DP, Pomès R (2011) Statistical convergence of equilibrium properties in simulations of molecular solutes embedded in lipid bilayers. J Chem Theory Comput 7: 4175-4188.

9. Scheidt HA, Huster D, Gawrisch K (2005) Diffusion of Cholesterol and Its Precursors in Lipid Membranes Studied by 1H Pulsed Field Gradient Magic Angle Spinning NMR. Biophys J 89: 2504-2512.

1. The number of DPPC and cholesterol molecules is not exactly the same in the 50% mol. case because while inserting the NP, lipids that were overlapping with the NP were deleted. [↑](#footnote-ref-1)
